# Lessons from Cancer Immunoediting in Cutaneous Melanoma

**DOI:** 10.1155/2012/192719

**Published:** 2012-08-14

**Authors:** Mariana Aris, María Marcela Barrio, José Mordoh

**Affiliations:** ^1^Laboratorio de Cancerología, Fundación Instituto Leloir, IIBBA-CONICET, Avenida Patricias Argentinas 435, 1405 Buenos Aires, Argentina; ^2^Centro de Investigaciones Oncológicas, Fundación Cáncer and Instituto Alexander Fleming, 1426 Buenos Aires, Argentina

## Abstract

We will revisit the dual role of the immune system in controlling and enabling tumor progression, known as *cancer immunoediting*. We will go through the different phases of this phenomenon, exposing the most relevant evidences obtained from experimental models and human clinical data, with special focus on Cutaneous Melanoma, an immunogenic tumor *per excellence*. We will describe the different immunotherapeutic strategies employed and consider current models accounting for tumor heterogeneity. And finally, we will propose a rational discussion of the progress made and the future challenges in the therapeutics of Cutaneous Melanoma, taking into consideration that tumor evolution is the resulting from a continuous feedback between tumor cells and their environment, and that different combinatorial therapeutic approaches can be implemented according to the tumor stage.

## 1. Introduction

Tumor transformation and progression depends on the cell type and its genetic and epigenetic modifications, where cells overpass several intrinsic tumor suppressor mechanisms and acquire distinctive and complementary capabilities allowing tumor growth and metastatic dissemination [1]. Also, it relies on the interaction of tumor cells with the surrounding environment, the stroma, and the overcoming of extrinsic tumor suppressor mechanisms. In this paper, we will focus on the complex interaction between cancer cells and the immune system, with both controlling and enabling functions, namely, the *cancer immunoediting* theory. In particular, we will discuss the case of Cutaneous Melanoma (CM), a prototypic immunogenic tumor, and include a critical overview of the different immunotherapeutic approaches employed so far.

## 2. Historical Perspective of the Cancer ****Immunoediting Theory

The idea that the immune system (IS) is involved in controlling tumor development and progression has been the subject of discussion for many years. In the XX century, Paul Ehrlich stated the theory of cancer immunosurveillance, reformulated in 1957 by Burnet and Thomas, which proposed that the IS is responsible for preventing tumor development in immunocompetent organisms [2]. They reasoned that cancer would be much more frequent in long-lived organisms if it were not for the action of the IS.

The role of IS in tumor control remained controversial until the development of improved genetically-modified murine models of immunodeficiency in the 1990s. Previously, the use of athymic nude mice has mistaken this concept, because no differences were found in tumor incidence between nude and immunocompetent wild type mice [3]. Nowadays, we know that nude mice are not fully immunodeficient, as they have NK cells and some extrathymic T-cell populations [4]. The first supporting evidence proceeded from a landmark work from Robert Schreiber's group, in which the role of IFN-*γ* in tumor surveillance was proven by demonstrating an increased incidence of chemically induced or spontaneously arising tumors in genetically-modified mice deficient for IFN-*γ* or all IFN receptors (Rc) (Stat-1-deficient mice), with respect to immunocompetent wild type mice [5]. Similar results were obtained for perforin in a model of spontaneous lymphoma, standing out the relevance of lymphocyte cytotoxicity (NK, NKT, and CD8 cells) in preventing tumor development [6]. Later on, the role of NK and NKT cells in protection against carcinogenesis was shown in different experimental models [7]. It was getting clear that mice that lacked components of the innate or the adaptive IS would have a dramatically increased rate of tumor formation. In this regard, additional experiments were performed, revealing that immunodeficient mice were more susceptible to carcinogens than immunocompetent mice [8]. Moreover, it was evidenced that the IS not only controls the number of tumor cells but also their immunogenicity, as tumors developed in immunodeficient mice were more immunogenic (unedited) than similar tumors developed in immunocompetent mice (edited). Therefore, the IS would be involved both in tumor development and in tumor edition of immunogenicity [8]. This stands for the theory of *cancer immunoediting*, where the IS has a dual role, both suppressing and enabling cancer. It can repress tumor growth by killing cancer cells or arresting proliferation, but it can also enable tumor growth, either by the selection of less immunogenic cells better adapted to survive in an immunocompetent host, or by the establishment of a tumor-permissive microenvironment that enables tumor growth.

## 3. The Mains of Cancer Immunoediting

Several experiments were performed in different immunodeficient murine models, where spontaneous as well as carcinogen-induced tumor development were analyzed, along with the study of immunodeficiency's effect on genetically engineered murine tumor models, which all support and contribute to describe the cancer immunoediting process (reviewed in detail in [9]). The cancer immunoediting theory postulates 3 phases that describe tumor evolution in light of its interaction with the IS: elimination, equilibrium, and escape [2]. Cancer cells communicate with stromal cells either by direct contact or by cytokine and chemokine signaling, proceeding in autocrine and paracrine ways to control and shape tumor growth. And it is the integration of all these signals along with the activation state of the different cell types in the tumor environment that determines whether the equilibrium is displaced to an antitumor response, or to a tumor-permissive environment.

### 3.1. Elimination

This is the immunosurveillance phase, in which both innate and adaptive immunity work together to detect and destroy tumor cells. This process is an extrinsic tumor suppressor mechanism that acts on cancer cells, in which intrinsic tumor suppressor mechanisms have already failed. In the beginning of tumor development, dying tumor cells and damaged-surrounding tissues release factors like IFN-*γ*, IFN-*α*/*β*, and DAMPs [10–12]. These signals recruit cells from innate (NK, NKT, *γδ* T cells, macrophages, and dendritic cells) and adaptive IS (CD4 and CD8 T cells). Tumor cells expressing NKG2D activate NK cells. Tumor infiltrating NK cells and macrophages activate each other by production of IFN-*γ* and IL-12, and kill tumor cells by apoptosis via TRAIL, perforins and reactive oxygen and nitrogen species. The activation of dendritic cells promotes the induction of an adaptive immune response, through tumor antigen (Ag) presentation to CD8 cytotoxic T cells (CTL) with help from CD4 cells, ideally generating a long-lasting immune response. Tumor Ag were first evidenced thanks to the finding that mice immunized with carcinogen-induced tumors were protected in case of a new challenge with the same tumor [13]. There are different types of *tumor Ag*, including those coded by aberrantly expressed normal genes (melanocyte differentiation Ag in CM); tumor-mutated genes (p53); cancer-testis genes, that in physiological conditions are only expressed in germ cells (MAGE and NY-ESO-1); and genes encoding viral proteins (HPV proteins).

If the tumor is completely destroyed by the IS, the elimination phase would complete cancer immunoediting. It is important for early tumor control its origin (spontaneous or induced by a carcinogen) as well as its anatomic localization and growth rate. Nowadays, we know that the IS prevents cancer development by different ways: it protects the host from viral infections; it prevents an inflammatory environment that enables tumorigenesis by abruptly removing pathogens; and it eliminates tumor cells by effector cells from the innate and adaptive IS.

In a recent controversial work where, oppositely to the traditional model of primary tumor progression to metastasis, it was proposed that tumor dissemination to secondary organs would be an early event upon transformation, but cancer cells would remain in a dormant state, resulting in staggered metastatic outgrowth [14]. In a murine model of spontaneous melanoma, tumor cells were found to disseminate early in the development of the primary tumor and remain dormant according to the tissue. Dormant cells from lung showed low proliferation rate in comparison to primary tumors, which was partly mediated by cytostatic CD8^+^ T cells. Therefore, immune strategies that favor the dormancy of disseminated cells can control the development of metastases.

### 3.2. Equilibrium

This phase takes place when a group of tumor cells survive the initial attack from the IS and move into an equilibrium phase, in which tumor cells are controlled by the IS but cannot be completely eliminated. In this way, tumors can be controlled by the IS for long periods of time, encompassing the host's entire life. T cells, IL-12, and IFN-*γ* are known to sustain the dormancy state [15].

Tumor cells may remain quiescent, with no cell division or apoptosis [16]; or may proliferate and become balanced by apoptosis, with no increase in number [17]. Continuous interaction of the tumor with the IS may lead to the edition of tumor immunogenicity, where cancer cells are modified, generating less immunogenic tumor variants that may escape control by the IS, proliferating and developing clinically detectable tumors.

An experimental model of equilibrium was established by administrating low doses of carcinogen MCA (3-methylcholanthrene) in wild type mice, which was interrupted when tumors arose after CD4, CD8, and IFN-*γ* depletion [15]. However, the same experiment performed in immunodeficient Rag^−/−^ mice did not introduce any change, meaning that disruption of equilibrium would not occur as a result of prolonged *de novo* transformation. Careful examination of the stable mass at equilibrium revealed the presence of atypical cells, with low proliferation index, that induced tumor formation when transplanted into immunodeficient mice. And edited cells from arising tumors were found to be less immunogenic than unedited cells from equilibrium. These experiments showed that cancer cells in equilibrium proliferate poorly and remain unedited, until they spontaneously become edited and escape immune control and grow.

### 3.3. Escape

Continuous pressure by the IS on genetically unstable cells can lead to the generation of tumor variants that (i) are no longer recognized by the IS, (ii) become insensitive to effector mechanisms, and (iii) induce an immunosuppressor, tolerant microenvironment. Also, changes in the IS are induced that might contribute to a tumor-permissive environment. As a consequence, tumors progress. In Table 1, several of the mechanisms involved in tumor escape are described, including changes in tumor cells and in IS cells, interfering especially with innate and cellular immune response. Supporting references are provided both by experimental models and clinical data from human patients. We will focus on CM, a prototype immunogenic tumor.

## 4. Cutaneous Melanoma: A Test Field ****for Immunotherapy

CM is the neoplasia originated from melanocytes that develops in the skin, and it has the fastest growing incidence worldwide [18]. At the clinical-histological level, the Clark model proposes a gradual transition from normal melanocytes to dysplastic nevi, then to primary CM, including radial and vertical growth phases, eventually leading to metastasis (mts) to the lymph nodes (LN) and distant organs [19]. Early diagnosed tumors (stages I-II, AJCC) are curable by surgery in more than 90% of cases; however, when CM metastasizes, only a minority of patients can be cured [20]. CM would not respond to conventional therapies like radiotherapy and chemotherapy; nevertheless, as it is an immunogenic tumor, it allows the use of immunotherapy as an alternative. Among the strongest supporting evidences for the dual role of the IS in CM eradication and progression are included the following:

**(a) Tumor Antigens**

The presence of tumor Ag in humans was shown by modern methodologies, involving the use of antibodies and CTL derived from patients as probes, tested on autologous tumor cell libraries. Among CM Ag outstands melanocyte differentiation Ag (MD-Ag) such as MART-1 [21, 22], gp100 [23], tyrosinase [24], tyrosinase-related protein-1 (TRP1) [25], TRP2 [26], and MELOE-1 [27]; cancer-testis Ag from the MAGE super-family [28] and NY-ESO-1 [29]; and tumor-mutated Ag such as BRAF [30].

**(b) Spontaneous regressions**

The finding of both humoral and cellular immunity to tumor Ag suggests that the IS is capable of eliciting a coordinated immune response to tumors as it would to a foreign Ag. Indeed, several tumor regressions were observed in patients as a consequence of the action of the IS [31]. Infiltration of IS cells is an early event in transformation and it is associated with disease outcome. There are several studies in CM patients that correlate quantity, quality, and distribution of tumor infiltrating lymphocytes (TIL) with patient survival [19, 32, 33]. The first studies just analyzed the presence and distribution of lymphocytes; more recent studies also focus on the immunophenotype of IS cells, as it is known that the IS may move from an antitumor environment to a tumor-permissive one. It was described in a case report a CM patient treated with anti-CTLA-4 therapy that was undergoing simultaneously the three phases of cancer immunoediting, with regressing, stable, and progressing lesions [34]. Probably, the environment of the different metastases (mts) would account for tumor response. In another clinical case, different rounds of immunoediting, escape and immune adaptation by shifting of the T-cell response were observed [35].

**(c) Immunodeficiency**

In general, immunodeficiency is associated with an increased risk of developing cancer. Most related factors include viral oncogenesis and reduced tumor immunosurveillance. Immunocompromised patients, like transplant recipients or AIDS patients, develop lymphomas (Epstein-Barr virus), Kaposi's sarcoma (Herpes virus), and cervical cancer (Human papillomavirus) [36]. An increased incidence of tumors non-related to virus, like colon, lung, pancreas, kidney, head and neck, skin carcinomas and CM was also observed [37]. In a case report, it was described that two patients that received kidney grafts from a common donor developed CM. It was further revealed that the donor had overcome this pathology in the past; therefore, the donor's kidney probably contained CM cells held in equilibrium by the IS. When kidneys were grafted into immunosuppressed recipients, the development of CM was favored [38]. These evidences are consistent with the idea that tumors progress in immunosuppressive permissive environments.

**(d) Immunosuppression**

Although CM is highly immunogenic, tumors develop and progress in immunocompetent patients. One of the contributing factors is the induction of a local state of immune suppression and tolerance to tumors as a result of tumor interaction with its environment. Cancer cells develop different mechanisms for tumor escape, including evasion of Ag recognition by the IS and secretion of immunosuppressor and proapoptotic factors (Table 1). Analysis of immunosuppressor factors in primary CM biopsies, negative and positive sentinel lymph nodes (SLN), and LN with advanced metastasis revealed that primary CM cells secreted TGF-*β*2 that renders dendritic cells tolerogenic; tolerogenic dendritic cells (tDC) and Treg were found at all stages, with increasing IDO and IL-10 secretion with CM progression, making the SLN an immunoprivileged site suitable for metastasis [39]. Thus, tumor cells would secrete immunosuppressor factors that would render IS effector cells into a tolerant phenotype, which in turn would secrete more immunosuppressor factors preparing the niche for metastasis before tumor dissemination. Recently, it was shown that secretion of CCL21 by melanoma cells promotes tolerance in syngeneic and xenograft CM models [40]. CCL21^low^ tumors presented specific CTL for CM Ag and cytokines related to an immunogenic response. Instead, CCL21^high^ tumors secrete TGF-*β*1, promote CCR7-dependent Treg and MDSC activation, and increased lymphoid tissue inducer cells, which promoted lymphoid neogenesis.

*Regulatory T cells* (Treg) are key immunosuppressor factors. The presence of CD4^+^CD25^+^FOXP3^+^ Treg was analyzed among different nevi (common/atypical junctional and compound nevi, Spitz nevi) and primary CM [41]. These regulatory cells were found in all these lesions, but were more represented in atypical junctional/compound nevi and in radial growth phase CM, suggesting that Treg induce immunotolerance early during CM genesis, favoring CM growth. Indeed, Indoleamine 2,3-dioxygenase (IDO) expression in Treg, an enzyme with immunosuppressive properties, was identified as a negative survival prognostic marker in SLN^−^ patients [42]. Moreover, tumor Ag-specific CD4^+^CD25^+^FoxP3^+^ Treg were evidenced in the blood of patients with metastatic CM [43]. These cells recognized a broad range of tumor Ag, including gp100, NY-ESO-1, TRP1 and inhibitor of apoptosis protein (IAP), and proliferated in response to specific peptides. They produced preferentially IL-10 and suppressed autologous CD4^+^CD25^−^ T-cell responses in a cell contact-dependent manner; they were not detected in healthy individuals. Therefore, these tumor-Ag-specific Treg might represent a target for improving CM immunotherapy.

*Plasmacytoid dendritic cells* (pDC) are characterized by the induction of strong immunosuppression. Both pDC and neutrophils were found associated with pSTAT-3 expression in CM, resulting in markers of poor prognosis in primary CM [44]. Also, pDC accumulate in SLN^+^ and express IDO, promoting immunotolerance [45, 46].

With regard to *effector cells*, a natural function of NK lymphocytes is to kill cells that fail to express MHC I molecules, thereby contributing to tumor eradication. The most frequent event observed in NK cells during CM progression is loss of activating Rc and increase of inhibitory Rc [47, 48]. With respect to CTL, the induction of an immunotolerant state interferes with the cytotoxic function of CTLs, as IFN-*γ* and perforin expression decrease [49, 50]. Also, tumor cells secrete factors, like Galectin-3, that induce apoptosis of CTL and NK cells [51, 52]. With regard to tolerance, the functional state of tumor-specific CTLs anti-MART-1 from peripheral blood and metastasis populations from CM patients was compared [49]. TILs expressed lower levels of IFN-*γ* and perforin than peripheral T cells, indicating a local state of tolerance. However, cytotoxic activity could be recovered after re-stimulation of CD8 cells by *in vitro* culture; therefore, local induction of tolerance would be reversible.

**(e) Inflammation**

Chronic inflammation is a key factor involved in tumor development and progression (reviewed in [53]). Sun exposure promotes an inflammatory environment in the skin, increasing the risk of developing skin cancer, including CM [54]. Inflammation contributes to tumor initiation by increasing the DNA mutation rate, and through production of reactive oxygen and nitrogen species that induce DNA damage and instability. Also, it activates *tissue repair responses*, inducing proliferation of premalignant cells and enhancing their survival. Tumor-infiltrating IS cells secrete cytokines that activate *key transcription factors* in transformed cells, like NF*κ*B or STAT-3, that control survival, proliferation, growth, angiogenesis, and invasion [55]. In turn, these transcription factors induce chemokines that attract additional inflammatory IS cells to sustain tumor-associated inflammation. Upon transformation, inflammation stimulates angiogenesis and causes local immunosuppression, helping tumor cells to survive and accumulate additional mutations as well as epigenetic changes, enabling tumor progression.

*Macrophages* are key mediators of the inflammatory response. Macrophages can be classified into M1 and M2 types [56]. *M1 macrophages* are associated with the acute inflammatory response, capable of killing pathogens, and priming antitumor immune responses. They can be activated by IFN-*γ* and pathogens and express high levels of pro-inflammatory cytokines (TNF-*α*, IL-1, IL-6, IL-12, or IL-23), MHC molecules and NO (nitric oxide) synthase. On the other hand, *M2 macrophages* (or “alternatively” activated macrophages), induced *in vitro* by IL-4, IL-10, and IL-13, downregulate MHCII and IL-12 expression and increase IL-10, scavenger receptor A, and arginase. This phenotype is related to an inflammatory tumor-permissive environment. However, M1 and M2 macrophages phenotype is plastic since it is defined by gene expression profiles; oppositely to CD4 T_*H*_1 and T_*H*_2 cells, which involved differentiation committed pathways. In CM, M2 *tumor-associated macrophages* (TAM) release tumor-enabling factors, including angiogenic and growth factors. Overexpression of monocyte chemoattractant protein (MCP-1) on CM cells attracted macrophages, enabling tumor growth and angiogenesis in a human xenograft model [57]. Also, adrenomedullin expression by TAM enables tumor growth and angiogenesis in CM B16 [58]. Analysis of TAM at different stages including benign nevi revealed more frequency of COX-2^+^ TAM in primary CM, proposing COX-2 as a marker of CM progression [59].

*Myeloid-derived suppressor cells* (MDSC), a heterogeneous group of progenitor and immature myeloid cells, have emerged as key immune modulators in various human malignancies. In several experimental models, it was shown that chronic inflammation recruits MDSC to the tumor, expressing immunosuppressor factors, impairing T-cell function and enabling metastases [60–62]. MDSC frequency is increased with CM progression, and STAT-3 is a key factor in MDSC development and function [63].

*Tumor-associated neutrophils* (TAN) were shown to promote CM cells migration via MAC-1/ICAM-1 interaction [64], and to be associated with poor prognosis at all stages [44, 65]. Finally, *mast cells* are also implied in tumor-associated inflammation. TNF-*α* and histamine secreted by mast cells induced expression of the inflammatory cytokine IL-8 by CM cells [66]. Tryptase^+^ mast cells were found related to VEGF expression, enabling angiogenesis in CM [67]; therefore, they were associated with poor prognosis for CM [68].

On this basis, different therapeutic approaches were employed with the main purposes of overcoming chronic inflammation, immunosuppression, and tolerance induced by the own tumor and its environment, and to stimulate tumor Ag immunogenicity and effector function of immune cells in order to eradicate tumors. We will discuss different examples, highlighting evidences from cancer immunoediting, and progresses and challenges in the treatment of CM.

### 4.1. Adjuvant Therapies

Among drugs used to stimulate immune effector cells, high-dose Interferon alfa2B (IFN-*α*2b) is a FDA-approved drug for use in patients with stages II-III CM. Whereas it increases disease-free survival and has a moderate effect on overall survival (OS), it is nevertheless related to severe side effects [69]. Another adjuvant therapy commonly employed, interleukin-2 (IL-2), promotes proliferation of T, B, and NK cells. This drug is approved for stage IV CM patients, with 16% objective response (OR) rate, including 6% complete response rate, although associated with short-term acute toxicity [70]. In a case report, a patient presented loss of the TAP-1 and MD-Ag MART-1 within subsequent metastases developed after several therapies, including IL-2 [71]. Cytogenetic analysis of the subsequent metastases revealed similar profiles, indicating a common genetic composition. Sensitivity to previous CTL clones could be restored by introducing MART-1 and TAP-1 by retroviral expression, further supporting the immunoediting of this tumor during its progression.

### 4.2. Molecular Target-Specific Therapies

Among target-specific therapies are the blockade of oncogenes. The most frequent mutation found in CM (50–65%) is a driver mutation in the BRAF oncogene, BRAF^V600E^, involved in the MAPK proliferation pathway [30]. Specific inhibitors were designed for BRAF^V600E^ and tested on advanced CM patients. In a phase III study, comparison of the BRAF^V600E^ inhibitor Vemurafenib with the chemotherapeutic drug Dacarbazine showed more than 50% of response rate to Vemurafenib, with a sensitive increase in overall survival and progression-free survival (PFS) in comparison to Dacarbazine [72]. This inhibitor allowed achieving important remissions, although transitory, because relapses were observed. However, the administration of Vemurafenib does not interfere neither with the viability nor functionality of T cells, allowing the implementation of a combinatorial approach with immunotherapy [73]. The increase in the flow of CD4 and CD8 cells to the tumor site after beginning of Vemurafenib administration further supports combinatorial strategies [74]. In a recent landmark work, in MYC^+^ and BCR-ABL^+^ lymphoma and leukemia mouse models of oncogene addiction, it was shown that CD4^+^ cells are involved in cellular senescence, shutdown of angiogenesis and chemokine expression [75]. This provides evidence that the IS plays a role in tumor regression upon oncogene inactivation, a process that was considered cell-autonomous, adding scientific rationale for combination therapeutic approaches.

### 4.3. Immune Tumor-Specific Therapies

The main routes employed to promote tumor-specific immunity include the active way through the use of *therapeutic vaccines* (*in vivo*), and the passive way through Adoptive Cell Therapy (ACT) (*ex vivo*). Therapeutic vaccines are administered after surgery with the purpose of mounting a long-lasting immunity and controlling any micrometastatic foci. The rational base of vaccines is that tumor Ag must be captured by dendritic cells, which migrate to lymph nodes to activate CD4 and CD8 cells, triggering an adaptive immune response. Therapeutic vaccines include tumor Ag vaccines, with different sources of Ag, like peptides, tumor lysates, recombinant virus, or whole irradiated cells; dendritic cells vaccines, consisting of autologous dendritic cells stimulated *in vitro* with a proper Ag source, maturated *ex vivo*, and then injected back into the patient. ACT involves the expansion of autologous T cells *ex vivo* to achieve sufficient number to eliminate important tumor masses. Either TILs or genetically modified T cells (with clonotypic TCR or chimeric antigenic receptor) might be perfused. This is an attractive approach for advanced patients whose tumors cannot be removed by surgery. However, the convenience of this expensive treatment has not yet been validated in randomized, prospective clinical trials.

With regard to tumor Ag vaccines, in a trial with MART-1, gp100 and tyrosinase peptides in metastatic CM patients, one of the patients who experienced a dramatic tumor regression had preexisting immunity to TRP2 and NY-ESO-1 Ag [76]. Of interest, the immune reactivity against TRP2 persisted over time, whereas that against NY-ESO-1 waned over the course of follow up; it is possible that residual tumors were immunoselected *in vivo* for loss of NY-ESO-1 over time. Other clinical cases of tumor regression upon vaccination with MAGE-1 peptide indicated a higher frequency of CTL towards general tumor Ag (antitumor CTL) than specific vaccine-Ag (anti-vaccine CTL), both in the tumor and in circulation [77, 78]. Antitumor T cells were already present in the patient before vaccination, with some highly dominant clonotypes. Thus, preexisting antitumor T cells may be ineffective at rejecting the tumor either because their frequency is too low, because tumor cells were selected to escape recognition, or because such lymphocytes are functionally deficient. However, this state of functional tolerance might be reversed by the administration of vaccines. A possible explanation is that vaccination induces cytokine cascades both locally and systemically, resulting in activation and proliferation of anti-melanoma Ag precursors, and infiltration of these effectors into tumors. Thus, a spontaneous antitumor T-cell response, which has become ineffective, can be reversed by vaccination and contribute to tumor rejection.

Actually, vaccination with tumor-Ag vaccines has been extensively assayed in CM patients, so far with little success [79, 80]. What could be the reasons for the failure of therapeutic vaccination in a large majority of the patients? A possible explanation is the low occurrence of anti-vaccine T cells that have the required functional properties to migrate to the tumor, resist the inhibitory tumor environment, and initiate focal activation. Another factor might be the inability to overcome the severe immunosuppressive environment, preventing the effectiveness of any vaccine. In fact, recent studies indicate that selective Treg depletion improves therapeutic effect of vaccines [81]. Another issue is that vaccines that target a unique Ag are in disadvantage, since tumors in general are heterogeneous, CM being not an exception. Therefore resistance may come from the coexistence of heterogeneous populations, or acquired by loss of Ag or HLA expression. In this regard, targeting multiple targets provides a step forward. In this way, previous clinical trials conducted by us indicated that vaccination with allogeneic irradiated cells, in patients in early stages of the disease, may prolong significantly disease-free survival [82]. Also, promising results were obtained for patients with stages II/III CM in a phase I study with autologous dendritic cells loaded *ex vivo* with allogeneic irradiated cells [83, 84].

With regard to ACT, there are also different strategies. Culture of TIL is not suitable for all patients because of technical issues (reviewed in [85]), but achieved 50% OR in different trials [86, 87]. This procedure requires a previous cycle of immunosuppression in order to suppress the endogenous immunosuppressive environment from patients. This OR increased to 72% with more severe immunosuppressive before treatment, but required hematopoietic stem cells transfusion afterwards [88]. Young TIL protocols introduced shorter culture times, although administered TILs were unselected; however, it achieved 50% OR in a phase II study [89]. A limitation of this approach is the requirement that patients have preexisting tumor reactive cells that can be expanded *ex vivo*. Genetically modified T cells are derived from patient's blood cells, therefore are more feasible to be obtained. Cells transduced with most frequent tumor-regression Ag, MART-1 and gp100, allowed to achieve for MART-1 12% OR [90]; and 30% and 19% OR for MART-1 and gp100-specific CTL in another phase I study, including remissions of brain metastases [91]. Studies with cells modified with chimeric antigenic receptors for CM therapy are on the way. The principal disadvantage of specific CTLs/genetically modified T cells is that they target a unique Ag, easing the development of resistance. In a phase I study of TIL immunotherapy, although half of the patients presented clinical responses, almost 60% showed evidence of immunoediting with loss of MART-1 or HLA-I [86]. In other phase-I studies, the effect of MART-1 specific CTL clones in advanced-stage patients was analyzed, with half of the responders showing loss of Ag expression [92, 93].

## 5. Cutaneous Melanoma Heterogeneity and ****Immune Response

One question that arises from the observation of limited clinical responses and remissions in Ag-targeted therapies is about the nature of tumor growth and heterogeneity observed in CM. Whether there are different proliferative populations hierarchically organized, with distinguishing Ag, or there are unstable populations with variable proliferative potential. The *cancer stem cell* (CSC) model proposes a cellular hierarchy within tumors in which, as in physiological tissues, only the minor CSC subset would have unlimited proliferative potential, being capable of self-renewal and generation of differentiated cells, accounting for the tumor mass [94]. Oppositely, the stochastic or clonal evolution model states that most cells would self-renew, accounting for tumor growth. A more dynamic model of *phenotypic plasticity* is gaining momentum, in which cells would have a proliferative potential variable in time [95]. This is an important issue, since recent publications described that CM CSC, selected by CD271 expression, would not express MD-Ag MART-1, gp100, and tyrosinase [96]. Also, CM CSC selected by ABCB5, would not express MART-1 [97]. In contrast, it was reported that one out of four CM cells developed tumors in NOD/SCID Il2rg^−/−^ mice without any previous selection [98]; and that phenotypic plasticity, even in CSC markers like CD271 or ABCB5, would be a source of heterogeneity in CM [95].

We were interested in the study of the expression of immunotherapy-relevant Ag in CM proliferative populations. In particular, we wanted to disclose if cells expressing MD-Ag have limited proliferative potential, thus allowing MD-Ag non-expressing clonogenic cells (CC) to survive immune effectors and repopulate the tumor; we also wanted to address if CC would be intrinsically resistant to CTL. We focused on MART-1 and gp100, since in HLA-A0201 patients (40% Latin-Americans), most TILs are directed against them, thus appearing to be the most frequent Ag involved in tumor regressions [99, 100]. We analyzed MART-1/gp100 and the proliferation marker Ki-67 expression in primary and metastatic CM biopsies, observing the coexistence of MART-1/gp100 expressing and non-expressing populations that proliferated competitively, with no differences between primary and metastatic tumors. However, cells with differential proliferative potential might replicate. Therefore, we analyzed MART-1, gp100, tyrosinase, and CD271 expression in colonies obtained from anchorage-independent growing CC of human CM cell lines. By 7 days, colonies displayed positive, negative, and mixed expression patterns. By 14 days, Ag were downregulated, suggesting Ag plasticity. We found that plasticity in MART-1 expression involves promoter methylation. We studied MART-1 and gp100 expression along time in CM growing clones, revealing that Ag levels varied with time without interfering with clonogenicity. Finally, CC MART-1/gp100 expressing cells were efficiently lysed by specific CTL. In conclusion, we found that MD-Ag or CSC marker CD271 expression would not interfere neither with proliferation nor clonogenicity, and CC expressing the proper Ag and HLA-class-I haplotype would not be intrinsically resistant to lysis by CTL. Since MD-Ag-expressing and non-expressing cells are proliferative and clonogenic, giving rise to colonies of thousand cells, both subpopulations should be considered as targets to eradicate tumors [101].

## 6. Conclusions

*What do we know about cancer immunoediting?* Extensive research in this field reveals that there is a continuous feedback between the tumor and its microenvironment that determines tumor fate. It is not a fixed interaction, but rather a dynamic one, where signals from cancer and surrounding cells are constantly modifying each other giving an integral response. However, tumor evolution is progressive, and its study provides the possibility to interfere with this process with different therapeutic approaches according to the tumor stage (*different stages; different approaches*).

*What have we learned about CM and immunotherapy?* CM is a prototypic immunogenic tumor, with spontaneous regressions described in patients and with several Ag identified. Due to its limited therapeutic options when it metastasizes, immunotherapy has emerged as a remarkable one. In clinical practice, Ag-targeted therapies, even with vaccines or CTL, have achieved modest success. Among contributing factors, the specific blockade of the tumor immunosuppressor environment is a high wall to climb but it is indeed necessary, preserving as much as possible the immune repertoire from the patient. Also, it is relevant to overcome chronic inflammation, which fosters genomic instability, immunosuppression, growth, and angiogenesis in tumors. With regard to tumor cell heterogeneity, it is important to discern whether Ag heterogeneity is due to the presence of differentiated cells with limited proliferative potential (CSC model), or to Ag plasticity independent from proliferation (phenotypic plasticity model), accounting for resistance and escape from immune effectors. We and others found Ag and phenotypic plasticity even in CSC markers. Thus, if Ag expression varies in time, immunotherapeutic approaches should point towards plasticity or multitargeting of Ag, so at least some responder T-cell clones with proper migration capability and resistance to inhibitory factors would be obtained. In advanced patients, the equilibrium phase has largely been displaced, with a highly tumor immunosuppressive environment, many times with unresectable tumors. However, exciting approaches have arisen from studies of CM biology, like the use of BRAF^V600E^ inhibitors; recently, the role of the IS upon oncogene inactivation was evidenced, providing support for combinatorial therapeutic strategies.

*What do we learn about cancer immunoediting for improving therapeutic strategies?* We learn that the cancer immunoediting process considers the tumor as an integral organ with different components, including cancer cells as well as stromal cells, and so should therapeutic approaches do. Certainly, the continuous study of CM biology and its environment will improve combinatorial therapeutic approaches to reach an equilibrium state or, best of all, achieve tumor eradication.

## Figures and Tables

**Table 1:** Mechanisms involved in tumor escape in Cutaneous Melanoma. Examples of changes in tumor and immune cells derived from experimental models (syngeneic and xenograft murine models, as well as *in vitro* human models); and from clinical data from patients are described.

	Mechanism	Description	Examples	
			Experimental models	Clinical data
	Evasion of Ag recognition by the IS	Loss of Ag expression; HLA-I loss; Ag plasticity; fails in Ag presentation	MART-1 loss after specific CTL treatment in xenografts [102]; TYR and TRP-2 loss in CM B16 [103]; HLA-I loss in xenografts [104, 105]; plasticity in MD-Ag and CD271 expression [101]	Tumor immunoediting in subsequent metastases [106]; MART-1 loss [107]; TAP-1 and MART-1 loss [71] *β*-2 microglobulin loss [108]; HLA-I, MART-1, and TYR loss [109]; gp100, TRP-2, SOX10, NY-ESO-1, and HLA-I loss [110]; MART-1/HLA-I loss after specific ACT [86]; plasticity in several Ag, including ABCB5 and CD271 [95]; plasticity in MD-Ag and CD271 expression [101]
Changes in tumor cells	Secretion of immunosuppressor factors	Factors interfering with NK, macrophages, DC, CD4 and CD8, function	HLA-G secretion in exosomes [111], NKG2DL downregulation [112], and HLA-E expression [113] impair NK/CD8 cytotoxicity. CCL21 expression induces lymphoid-like stroma, and recruits/activates Treg and MDSC in syngeneic and xenografts models [40]	IDO expression recruits Treg enabling mts [114, 115]; ICOS-L promotes activation and expansion of Treg [116]; IL-10 expression recruits Treg & TAM and is associated with CM progression [117]; TGF-*β*1 expression recruits tDC & Treg [39]; IDO, TGF-*β*, and CCL17/CCL22 expression in genome-wide association studies [118];
	Secretion of proapoptotic factors	Factors inducing apoptosis in effector T cells (*tumor counterattack*)	FasL expression [119], PD1L (B7-H1) expression [120], and FASL and TRAIL expression induces T-cell apoptosis [121, 122]	Gal-3 expression induces apoptosis in TIL [51]; Gal-3 is a marker of progression [123]; Gal-1 & Gal-3 expression in genome-wide association studies [118]

Changes in IS cells	Natural killer cells (NK cells)	Impaired effector function	Gal-3 impairs NK cytotoxicity in CM B16 [52]	Impaired lytic granule polarization by HLA-G [113, 124]; loss of activating Rc CD161 & NKG2DR expression in CM mts [125]; loss of activating Rc (NKG2DR), increase of inhibitory Rc (CD158b), and impaired activity (lower CD107a, IFN-*γ* and TNF-*α* expression) in CM mts [47, 48]
	Cytotoxic T cells (CTL)/CD8 cells	Impaired effector function	TCR zeta-chain downregulation [126]	Decreased expression of IL-2, IL-4 and IFN-*γ* by TIL [127]; tolerance induction in mts, with loss of IFN-*γ* and perforin expression [49]; low proportion of CD8^+^CD27^−^ cytolytic cells in primary CM [50]
	Regulatory T cells (Treg)	They secrete immunosuppressive factors interfering with NK, macrophages, DC, CD4, and CD8 function	Treg inhibit FasL-induced innate and adaptive tumor immunity in CM B16 [128]	Induce immunotolerance in CM genesis [41]; upregulated in LN^+^ [129] and advanced CM [130]; Treg impairs NY-ESO-1 vaccine [131, 132]; secrete IL-10 [39]; Treg IDO^+^ as a negative survival prognostic marker for SLN^−^ patients [42]
	Tolerogenic dendritic cells (tDC)	tDCs have diminished Ag presentation and T-cell activation; induce anergy and tolerance in T-cells	P38 MAPK expression drives tDC in CM progression [133]	Upregulation in advanced patients [130]; TGF-R*β*I promotes tDC, expressing IDO and TGF-*β*2 [39]
Changes in IS cells	Plasmacytoid dendritic cells (pDC)	Induce strong immunosuppression	IDO expression and strong immunosuppression in mouse tumor-draining LN [134]	Related to poor prognosis in primary CM [44]; CCR6/CCL20 interaction involved in pDC recruitment to the tumor [135]; pDC associates with SLN^+^ [45] and express IDO [46]
	Tumor-associated macrophages (TAM)	M2-polarized TAM release immunosuppressor, proangiogenic, and growth factors	MCP-1 recruits TAM [57]; Adrenomedullin expression by TAM [58] is related to CM angiogenesis and growth.	Cyclooxygenase-2 expression in TAM as a marker of CM progression [59]; TAM impairs NY-ESO-1 vaccine [131]
	Myeloid-derived suppressor cells (MDSC)	Progenitor and immature myeloid cells induce Treg; aminoacids depletion; TCR modification	Treg recruits MDSC and induces B7-H1 and IL-10 expression in CM [60]; chronic inflammation recruits MDSC to tumor and impairs T-cell function [61]; MDSC enables CM mts [62]	Upregulated in advanced CM, MDSC immunosuppress CD4 and CD8 function [63]
	Tumor-associated neutrophils (TAN)	Promote chronic inflammation and tumor migration	Mac-1 (TAN)/ICAM-1 interaction promotes CM cells migration [64]	TAN associated with poor prognosis in primary CM [44], and an independent negative OS factor in advanced CM patients [65]
	Mast cells	Promote chronic inflammatory environment and angiogenesis	Il-8 inflammatory cytokine secretion by CM cells induced by mast cells [66]	Mast cells (tryptase^+^) association with VEGF expression and angiogenesis in CM [67]; mast cells correlation with poor CM prognosis [68]

## Abbreviations

ACT: Adoptive cell therapy
Ag: Antigen/s
CSC: Cancer stem cells
CC: Clonogenic cells
CM: Cutaneous melanoma
IDO: Indoleamine 2,3-dioxygenase
IS: Immune system
LN: Lymph node
mts: Metastasis/metastases
MDSC: Myeloid-derived suppressor cells
pDC: Plasmacytoid dendritic cells
PFS: Progression-free survival
OR: Objective response
OS: Overall survival
SLN: Sentinel lymph node
Rc: Receptor
TAM: Tumor-associated macrophages
TAN: Tumor-associated neutrophils
tDC: Tolerogenic dendritic cells
TIL: Tumor-infiltrating lymphocytes.
